# A Hierarchical RF-XGBoost Model for Short-Cycle Agricultural Product Sales Forecasting

**DOI:** 10.3390/foods13182936

**Published:** 2024-09-17

**Authors:** Jiawen Li, Binfan Lin, Peixian Wang, Yanmei Chen, Xianxian Zeng, Xin Liu, Rongjun Chen

**Affiliations:** 1 School of Computer Science, Guangdong Polytechnic Normal University, Guangzhou 510665, China; lijiawen@gpnu.edu.cn (J.L.); linfanfire@gmail.com (B.L.); wangpeixian@gpnu.edu.cn (P.W.); chch1980@163.com (Y.C.); 2 Guangxi Key Lab of Multi-Source Information Mining & Security, Guangxi Normal University, Guilin 541004, China; 3 Guangdong Provincial Key Laboratory of Intellectual Property and Big Data, Guangdong Polytechnic Normal University, Guangzhou 510665, China; 4 Guangdong Provincial Key Laboratory of Big Data Computing, The Chinese University of Hong Kong, Shenzhen (CUHK-Shenzhen), Shenzhen 518172, China; 5 Department of Electrical and Computer Engineering, University of Macau, Macau 999078, China; yb87445@um.edu.mo

**Keywords:** RF-XGBoost, hierarchical clustering, agricultural product, sales forecasting, food waste reduction

## Abstract

Short-cycle agricultural product sales forecasting significantly reduces food waste by accurately predicting demand, ensuring producers match supply with consumer needs. However, the forecasting is often subject to uncertain factors, resulting in highly volatile and discontinuous data. To address this, a hierarchical prediction model that combines RF-XGBoost is proposed in this work. It adopts the Random Forest (RF) in the first layer to extract residuals and achieve initial prediction results based on correlation features from Grey Relation Analysis (GRA). Then, a new feature set based on residual clustering features is generated after the hierarchical clustering is applied to classify the characteristics of the residuals. Subsequently, Extreme Gradient Boosting (XGBoost) acts as the second layer that utilizes those residual clustering features to yield the prediction results. The final prediction is by incorporating the results from the first layer and second layer correspondingly. As for the performance evaluation, using agricultural product sales data from a supermarket in China from 1 July 2020 to 30 June 2023, the results demonstrate superiority over standalone RF and XGBoost, with a Mean Absolute Percentage Error (MAPE) reduction of 10% and 12%, respectively, and a coefficient of determination (R^2^) increase of 22% and 24%, respectively. Additionally, its generalization is validated across 42 types of agricultural products from six vegetable categories, showing its extensive practical ability. Such performances reveal that the proposed model beneficially enhances the precision of short-term agricultural product sales forecasting, with the advantages of optimizing the supply chain from producers to consumers and minimizing food waste accordingly.

## 1. Introduction

China is home to over 18% of the global population, while its share of arable land is a mere 7% of the world’s total [1]. This imbalance between population and arable land has led to an ongoing widening gap between food supply and demand, indicating the persistent challenge of potential food shortages. Nonetheless, the situation is further complicated due to a critical issue of food waste in China, which translates to an annual waste of approximately 460 million tons of food [2]. Particularly alarming is the waste rate of staple agricultural products such as vegetables, grains, and fruits, which reaches up to around 30%, markedly higher than the 5% waste rate in Western countries [3]. Such a level of waste incurs not only considerable economic costs, estimated at 1.88 trillion Chinese Yuan (CNY) annually, but also poses increased resource depletion and environmental stress [4]. Thus, the timely forecasting of market demand for agricultural products is imperative, as it optimizes inventory management, reducing spoilage and enhancing product freshness. Efficient forecasting streamlines supply chains, leading to better coordination and reduced environmental impact. Additionally, it results in cost savings for producers and decision-makers and enhances consumer satisfaction by consistently providing fresh products, which is meaningful for deciding effective and sustainable food market-oriented strategies.

Usually, the relationship between agricultural product sales, market demand, prices, and production is intricate and dynamic. Market demand typically has a positive correlation with sales, where increasing demand leads to higher sales volumes. Nevertheless, this relationship is influenced by several factors, such as consumer purchasing power, seasonal variations, and the broader economic environment. Price, in particular, plays a vital role in affecting sales. Generally, as prices rise, sales tend to decline, especially for non-essential goods where consumers may seek substitutes. Conversely, for essential goods, the price elasticity is lower, meaning that price increases have a lesser impact on sales and, in certain cases, may even increase purchasing urgency. In this regard, accurate market demand forecasting enables producers to plan planting and production schedules aligned with market demand, preventing both overproduction and shortages [5]. Effective resource allocation through forecasting reduces production costs, minimizes waste, and improves overall production efficiency. Decision-makers can use forecasting data to adjust inventory and procurement strategies in advance, ensuring the timely availability of agricultural products and avoiding losses from stockouts or excessive inventory [6]. A more streamlined supply chain enhances operational efficiency, reduces energy consumption and transportation costs, and minimizes food waste, aligning with sustainable development goals. For consumers, accurate demand forecasting ensures access to fresh, high-quality agricultural products at reasonable prices [7]. This not only improves the overall shopping experience but also mitigates inconveniences associated with product shortages or price volatility. Therefore, accurate market demand forecasting is vital for maintaining market equilibrium and stability by optimizing supply chain management, refining inventory strategies, and informing government policy adjustments. It offers significant benefits to producers, decision-makers, and consumers.

In recent years, the rapid development of Artificial Intelligence (AI) with advanced machine learning algorithms has contributed to short-cycle agricultural product sales forecasting [8]. Technically, forecasting necessitates the consideration of a multitude of factors, including economic indicators, governmental policies, and climatic conditions, which involve analyzing the influence and underlying logical connections of these factors on agricultural yields and market prices [9]. Additionally, integrating historical data on production and pricing trends along with developmental patterns beneficially helps to attain accurate predictions for future agricultural output and sales [10]. Hence, data modeling, in an appropriate way, has emerged as a prevalent tool for predictive endeavors in related fields. 

Specially, several solutions have been proposed previously. For example, using Random Forest (RF), Olivares et al. [11] adopted weekly production data of banana bunches and Black Sigatoka (BS) epidemiological parameters from three adjacent banana sites in Panama between 2015 and 2018 to predict the number of banana bunches. An average variance of 70.0% and a Root Mean Square Error (RMSE) of 1107.93 ± 22 kg/ha were found. Therefore, the results validated that RF is an effective machine-learning method for predicting fruit products. Mishra et al. [12] applied and evaluated an Autoregressive Integrated Moving Average (ARIMA) model on India’s annual pulse production from 1961 to 2019. The assessment indicated that the ARIMA beneficially captures the time trend of pulse production and plays a key role in determining the gap between production and demand. Kuradusenge [13] et al. collected weather data and crop yields for potatoes and maize in Ireland and conducted prediction using an RF model. The results indicated that the RMSEs for potatoes and maize are 510.8 t/ha and 129.9 t/ha, respectively. In addition, compared to polynomial regression and the Support Vector Machine (SVM), they claimed that the RF demonstrates better performance, validating its suitability in food sales prediction. Paul et al. [14] estimated wholesale prices of eggplants in seventeen major markets in Odisha, India, using the General Regression Neural Network (GRNN) and compared it with Support Vector Regression (SVR), RF, and gradient boosting machine models. The evaluation indicated that the predictions from the GRNN model are closer to the actual prices, performing better than the other models. Haider et al. [15] used a Long Short-Term Memory (LSTM) model to predict wheat yields in Pakistan. The results revealed that Pakistan’s wheat production will gradually increase over the next 10 years. But, the production-to-demand ratio will continue to decline, which could pose a threat to the overall economy. Yin et al. [16] applied STL-Attention-based LSTM, which combines the Seasonal Trend decomposition of the Loess (STL) preprocessing method and the attention mechanism based on LSTM for vegetable sales forecasting. They also compared the proposed STL-ATTLSTM with three benchmarks (LSTM, attention-based LSTM, and STL-LSTM) and displayed that the STL-ATTLSTM can address the prediction lag issue caused by high seasonality.

Extreme Gradient Boosting (XGBoost) is often employed for prediction problems and has achieved good results. For instance, Gono et al. [17] used XGBoost to predict silver prices, accomplishing a Mean Absolute Percentage Error (MAPE) of 6.06% and an RMSE of 1.6967 US dollars. Wu et al. [18] utilized Particle Swarm Optimization (PSO) to optimize key parameters of the XGBoost model and then analyzed Australia’s electricity price data. They claimed that the optimized XGBoost properly adapts to the time-series trends. Tian et al. [19] designed a model named LSTM-BO-XGBoost with a Bayesian Optimization (BO) and applied it to stock price prediction. They validated that this model exhibits better stability than the other LSTMs, yielding RMSE, Mean Absolute Error (MAE), accuracy, and F1 score of 610.35, 15.60, 0.60, and 0.75, respectively. 

Moreover, ensemble learning has been used to enhance forecasting performance in a hybrid manner. For example, Gu et al. [20] proposed a Dual-Input Attention Long Short-Term Memory (DIA-LSTM) model for agricultural product sales forecasting. They accomplished an improvement in terms of MAPE from 2.8% to 5.5% over traditional models. Danandeh Mehr et al. [21] combined the Genetic Algorithm (GA) with RF to create a hybrid decision tree model called GARF. It employs various decision tree ensemble techniques and realizes good performance for predicting multi-temporal drought indices at two meteorological stations, Beypazari and Nallihan, in Ankara, Turkey. Fan et al. [22] integrated RF, SVM, and grey Verhulst models to forecast the electricity load of operators in Australia. The results displayed that the MAPE is 6.35%, which helpfully supports predicting electricity consumption. 

Generally, the fluctuation of product sales is influenced by production and demand, as well as by factors such as local policies, lifestyle habits, climate conditions, public opinions, and sales decisions. These factors are uncontrollable and difficult to collect, making short-cycle agricultural product sales forecasting a challenging task. In such circumstances, the availability of historical sales data and the underlying relationships make time-series analysis a powerful tool for predicting agricultural product sales. By analyzing historical sales data, patterns such as cyclic variations, trends, and seasonal influences can be identified, which allows the establishment of data models to predict future sales, promoting a reduction in resource use and waste generation throughout the distribution chain.

As for forecasting modeling, XGBoost performs well with structured data due to its unique tree-based structure. However, it often lacks flexibility in addressing time-series forecasting problems like short-cycle agricultural product sales. On the other side, RF captures nonlinear relationships or correlations within the time-series data. Specifically, it demonstrates robustness in handling time-series data forecasting. Based on that, to mitigate the uncertainty of individual models while better adapting to the dynamic and volatile nature of daily market demand for short-cycle agricultural products, this work proposes a hierarchical clustering model with the help of RF-XGBoost, aiming to achieve effective forecasting.

The rest of this work is organized as follows: Section 2 describes the experimental data evaluated in this work, as well as the data prepossessing. Section 3 presents the proposed method, offering detailed descriptions for each step. Section 4 discusses the results and conducts a comparative study with other approaches, aiming to validate the superiority of the proposed RF-XGBoost model. Finally, Section 5 shows the conclusion of this work.

## 2. Experimental Data

**Figure 1:**
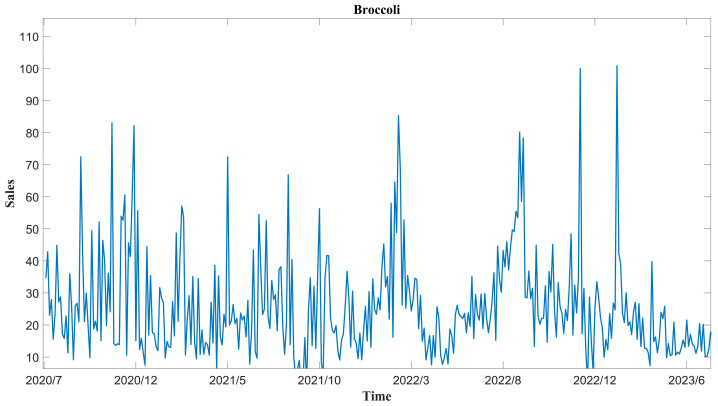
Broccoli sales volume data (kg) from 1 July 2020 to 30 June 2023.

The experimental data evaluated in this work comprises sales transaction details distributed by a supermarket in China from 1 July 2020 to 30 June 2023. The vegetable categories include leafy vegetables, peppers, solanacea, edible fungi, aquatic root vegetables, and cauliflower, totaling 246 types of individual product sales records from six vegetable categories. In these records, 42 short-cycle agricultural products with sales durations exceeding 100 days are selected. To facilitate reproducible research and make a positive effect on the academic field, the experimental data and source codes related to this work are freely available at https://github.com/fire-xian/Hierarchical-prediction (accessed on 15 July 2024), and as an example, the Broccoli sales data (kg) from 1 July 2020 to 30 June 2023, is drawn in Figure 1.

Concerning the workflow, initially, the 246 product sales records are separated, with each exclusively recording the sales records of the same product type. Subsequently, the sales volume of the same product on the same day is obtained, considering days as the unit of measurement. To simplify the study and account for the uncertainty in discount sales of short-cycle products, the impact of discounts on sales volume is disregarded in this work, and the average selling price of the same product on the same day is employed as the unit price for that product. The reason is that before conducting machine learning, selecting appropriate inputs is essential, which not only quantifies the correlation between different independent and dependent variables but also aids in interpreting the prediction results. The samples of short-cycle agricultural product sales data are shown in Table 1.

## 3. Proposed Method

### 3.1. Overall Framework

**Figure 2:**
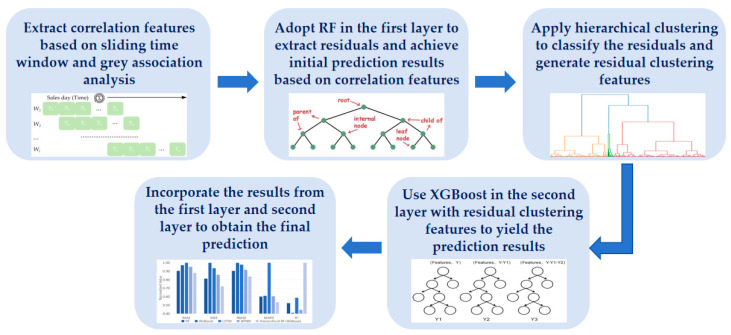
The overall framework of the proposed method.

For better illustration, Figure 2 depicts the overall framework of the proposed method. First, the sliding time window and Grey Relation Analysis (GRA) are utilized for preprocessing the sales data, aiming to extract the correlation features that show strong correlations with the target product. Then, RF is adopted in the first layer of the model to achieve initial prediction results based on the correlation features and extract the residual features derived from predicted and actual values. Next, hierarchical clustering is applied to classify the characteristics of the residuals and generate residual clustering features, denoted as a new feature set. Subsequently, XGBoost is involved in the second layer of the model, which randomly divides the new feature set into 60% training and 40% testing and yields the prediction results. After that, to incorporate the results from the first layer and second layer, the final forecasting can be obtained. More details are described in the following subsections.

### 3.2. Correlation Features Extraction

Correlation is useful for analyzing the relationships among individual products within various categories, as sales of one product usually drive the sales of related products, and focusing on such relationships is essential for extracting valuable features to enhance forecasting performance, which is also a systematic way to minimize the food waste [23]. However, in real-world scenarios, short-cycle agricultural product sales may not occur continuously due to the season, weather, logistics, or supply. To maintain the temporality of sales, the sliding time window is employed. After that, GRA is adopted to identify collaborative or competitive relationships among different products and obtain the correlation features correspondingly. 

**Figure 3:**
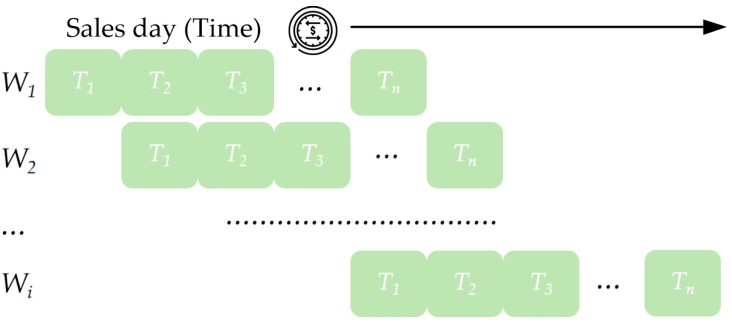
Sliding time window to fill in the missing values of product sales records.

**Figure 4:**
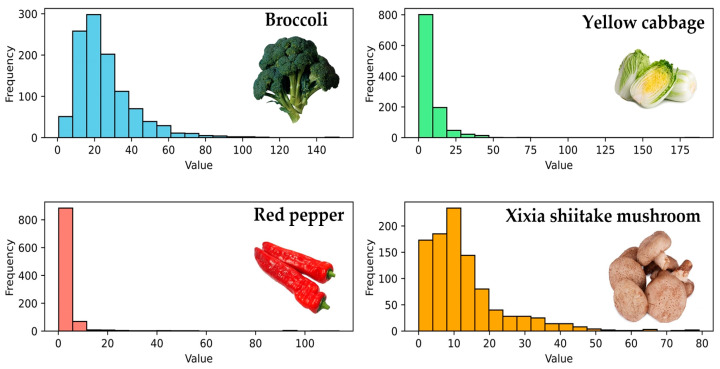
Histograms of the short-cycle agricultural product sales volume.

Using the sliding time window to handle missing values can smooth fluctuations and maintain the continuity of the time-series data, which beneficially improves model stability [24]. To obtain key changes in product sales over time, a sliding window is applied to segment the data, and the mean sales value of each segmented window is utilized to fill the missing values [25]. To this end, the information from multiple periods (*T*_1_, *T*_2_, *T*_3_, ..., *T_n_*) is employed and set as a time window (*W*_1_, *W*_2_, ..., *W_i_*) as illustrated in Figure 3, which slides forward to fill in the missing values correspondingly. In addition, to further investigate the distribution characteristics of the sales volume for choosing an appropriate correlation analysis method, the statistical histograms are drawn in Figure 4.

The histograms in Figure 4 indicate that the agricultural product sales do not exhibit the normal distribution. The reason is likely due to the influence of market demands that fluctuate over time and usually lead to cyclical or seasonal sales. In addition, food marketing strategies, such as promotions, advertising, and pricing, can cause sales to concentrate on specific periods. Consequently, in subsequent correlation analyses, it is improper to adopt methods like Pearson correlation, while GRA is suitable.

GRA explains the degree of correlation between different data as they change over time or across different objects [26]. If the trend of the two curves is consistent, indicating a high degree of synchronization, the correlation between them is considered high; conversely, it is low. Thus, GRA determines the closeness of relationships through the geometric similarity of sequence curves. It does not require data to follow a normal distribution and is applicable regardless of sample size or regularity, minimizing discrepancies between quantitative and qualitative analysis [27]. Technically, the GRA is performed by the following steps:

First, the reference and comparison series are identified. Let *X*_0_ be the reference series and *X*_0_(*i*) denote the *i*-th element in the *X*_0_ sequence, then *X_i_* (*i* = 1, 2, ..., *n*) is the comparison series, and *X_i_*(*i*) is the *i*-th element in the *i*-th subsequence, each sequence contains *m* elements:

(1) $$X_{0}=\left(X_{0}\left(1\right),X_{0}\left(2\right),\;\ldots ,X_{0}\left(m\right)\right)$$

(2) $$\left(X_{1},X_{2},\ldots ,X_{n}\right)=\left(\begin{array}{cc} \begin{array}{cc} X_{1}\left(1\right) & X_{2}\left(1\right)\\ X_{1}\left(2\right) & X_{2}\left(2\right) \end{array} & \begin{array}{cc} \cdots & X_{n}\left(1\right)\\ \cdots & X_{n}\left(2\right) \end{array}\\ \begin{array}{cc} \vdots & \vdots \\ X_{1}\left(m\right) & X_{2}\left(m\right) \end{array} & \;\begin{array}{cc} \vdots & \vdots \\ \cdots & X_{n}\left(m\right) \end{array} \end{array}\right)$$

Then, due to the diverse physical meanings of factors within the system, data may have different dimensions, making comparisons challenging or leading to incorrect results. Therefore, it is necessary to normalize the data using the mean method for dimensionless processing. Let *X_i_*(*k*) represent the *k*-th element in the *i*-th sequence, and ${\bar{X}}_{i}\left(k\right)$ denote the mean of the *i*-th sequence:

(3) $${\bar{X}}_{i}\left(k\right)=\frac{X_{i}\left(k\right)}{\frac{1}{m}\sum_{k=1}^{m}X_{i}\left(k\right)}$$

(4) $$\left({\bar{X}}_{1},{\bar{X}}_{2},\ldots ,{\bar{X}}_{n}\right)=\left(\begin{array}{cc} \begin{array}{cc} {\bar{X}}_{1}\left(1\right) & {\bar{X}}_{2}\left(1\right)\\ {\bar{X}}_{1}\left(2\right) & {\bar{X}}_{2}\left(2\right) \end{array} & \begin{array}{cc} \cdots & {\bar{X}}_{n}\left(1\right)\\ \cdots & {\bar{X}}_{n}\left(2\right) \end{array}\\ \begin{array}{cc} \vdots & \vdots \\ {\bar{X}}_{1}\left(m\right) & {\bar{X}}_{2}\left(m\right) \end{array} & \;\begin{array}{cc} \vdots & \vdots \\ \cdots & {\bar{X}}_{n}\left(m\right) \end{array} \end{array}\right)$$

In addition, the absolute differences between corresponding elements of the reference series and the comparison series are calculated by the following:

(5) $$|{\bar{X}}_{0}\left(k\right)-{\bar{X}}_{i}\left(k\right)|\left(k=1,2,\ldots ,m\right)\left(i=1,2,\ldots ,n\right)$$

After that, the maximum and minimum elements in all the sequences can be determined by

(6) $$min\left(min\left({|\bar{X}}_{0}\left(k\right)-{\bar{X}}_{i}\left(k\right)|\right)\right)\left(k=1,2,\ldots ,m\right)\left(i=1,2,\ldots ,n\right)$$

(7) $$max\left(max\left({|\bar{X}}_{0}\left(k\right)-{\bar{X}}_{i}\left(k\right)|\right)\right)\left(k=1,2,\ldots ,m\right)\left(i=1,2,\ldots ,n\right)$$

Lastly, let *ρ* = 0.5 to be the resolution coefficient, then the correlation coefficient $\varepsilon_{i}\left(k\right)$ between the reference series and the comparison series can be acquired by

(8) $$\varepsilon_{i}\left(k\right)=\frac{min\left(min\left({\bar{X}}_{0}\left(k\right)-{\bar{X}}_{i}\left(k\right)|\right)\right)+\rho max\left(max\left({\bar{X}}_{0}\left(k\right)-{\bar{X}}_{i}\left(k\right)|\right)\right)}{|{\bar{X}}_{0}\left(k\right)-{\bar{X}}_{i}\left(k\right)|+\rho max\left(max\left({|\bar{X}}_{0}\left(k\right)-{\bar{X}}_{i}\left(k\right)|\right)\right)}$$

The collaborative or competitive relationships between various products are identified based on the obtained correlation coefficients. Specifically, the correlation coefficients measure the degree of mutual influence between two products. When the coefficient is high, it indicates a collaborative relationship, meaning their sales mutually promote or closely affect each other. Conversely, when the coefficient is low, it reveals a competitive relationship, implying that the growth in sales of one product leads to a decrease in sales of another product. Such relationships are primarily due to similarities or significant differences in the market positioning of various products. Based on that, the correlation coefficients are valuable for sales forecasting. 

### 3.3. RF-Based First Layer

RF is a machine learning algorithm to solve classification and regression problems [28]. Particularly, it is a non-parametric ensemble learning method composed of the decision tree generated by reordering training data, where ensemble learning aims to improve model accuracy by combining the classification or prediction results of multiple models. RF initially creates several decision trees from the training dataset and then combines their outputs to obtain more accurate classifications or predictions. The output of RF is determined using majority voting. Due to the randomness, it is less prone to overfitting and exhibits strong forecasting capability when dealing with prediction problems [29]. 

In this work, RF is adopted in the first layer of the model. Then, the correlation features are randomly split into a training set (50%) and a testing set (50%), and sales volume is selected as the target variable. After that, the RF is trained utilizing the training set, and predictions are made on the testing set. Finally, the initial prediction results based on the correlation features are acquired, and the residuals between the predicted and actual values are calculated, which extracts residual features used in the next stage.

### 3.4. Hierarchical Clustering

Hierarchical clustering is a method employed to progressively merge or split objects in a dataset into clusters based on their similarity or distance. It builds a clustering structure that is represented by applying a dendrogram, revealing the hierarchical relationships between objects [30]. After obtaining the residual features from the first layer, hierarchical clustering is adopted to cluster these residuals. This process helps present the similarity between different residuals and highlights the characteristics of the residuals. Such characteristics are meaningful for generating the residual clustering features in the second layer.

To cluster the residual features, the pairwise distances between each pair of residual features are first calculated. Typical similarity measure includes Euclidean Distance, Canberra Distance, Manhattan Distance, and so on [31]. In this work, the hierarchical clustering adopts Euclidean distance as the similarity measure. Suppose to $x=\left(x_{1},x_{2},\ldots ,x_{n}\right)$ and $y=\left(y_{1},y_{2},\ldots ,y_{n}\right)$ are two points in an *n*-dimensional space, the Euclidean distance $d\left(x,y\right)$ can be expressed as

(9) $$d\left(x,y\right)=\sqrt{{\left(x_{1}-y_{1}\right)}^{2}{+\left(x_{2}-y_{2}\right)}^{2}+\ldots +{\left(x_{n}-y_{n}\right)}^{2}}$$

Subsequently, there are two strategies in hierarchical clustering: agglomerative (bottom-up) and divisive (top-down) [32]. Agglomerative clustering initiates with individual data points and gradually merges the closest clusters into larger clusters until all data points are merged into one big cluster. Divisive clustering starts with a single large cluster containing all data points and gradually splits it into smaller clusters until each cluster contains only one data point. Here, the agglomerative approach is used, as it is more appropriate for sales data property. Additionally, during the process, a clustering criterion needs to be defined to determine how clusters are merged or split [33]. Minimizing the variance of the merged clusters is employed as the clustering criterion in this work. As a result, the residual features can be classified into three categories and represented as residual clustering features. 

### 3.5. XGBoost-Based Second Layer

XGBoost is a method based on ensemble learning and implemented as an optimized library built on the Gradient Boosting Decision Tree (GBDT). XGBoost sequentially creates multiple decision trees, each aiming to reduce the error of the previous one. In each iteration, XGBoost trains a new decision tree by fitting the residuals, gradually reducing the model’s error accordingly. The unique tree structure of the XGBoost model enables it to perform well with structured data, although it may lose flexibility when addressing time-series forecasting problems [34]. 

In this work, the XGBoost is employed as the second layer of the model. A random factor is involved, and the new feature set of residual clustering features from previous operations is split into a training set (60%) and a testing set (40%), and sales volume is the target variable. The XGBoost is trained to predict the testing set, and based on the results through the XGBoost in the second layer, the final prediction results are yielded by summing them with the corresponding predictions from the first layer. 

## 4. Results and Discussion

### 4.1. Evaluation Metrics

To assess the method performance and validate the results, MAE, MAPE, Mean Squared Error (MSE), RMSE, and the coefficient of determination (R^2^) are used as evaluation metrics, which provide different perspectives to assess the quality of the models. Therefore, by comparing them across different models, the model performance in fitting and predicting the sales data can be found.

MAE is the average absolute error between the predicted and actual values. It assigns equal weight to each error, avoiding the amplification of large errors. The larger the MAE, the less accurate a model is at predicting the target values. Thus, a higher MAE indicates that the predictions are, on average, further from the actual values. Conversely, a lower MAE indicates that the predictions are closer to the actual values, revealing a more accurate model. Suppose *n* is the number of data points, $y_{i}$ is the *i*-th actual value, and ${\hat{y}}_{i}$ is the *i*-th predicted value, MAE is obtained by

(10) $$MAE=\frac{1}{n}\sum_{i=1}^{n}\left|\left(y_{i}-{\hat{y}}_{i}\right)\right|$$

MAPE is the average of the absolute percentage error between the predicted and actual values. It is a measure used to assess the accuracy of a forecasting model and expresses the error as a percentage of the actual values. A high MAPE indicates low accuracy, meaning the model’s predictions are far from the actual values and vice versa. MAPE is expressed as

(11) $$MAPE=\frac{100\%}{n}\sum_{i=1}^{n}\frac{\left|y_{i}-{\hat{y}}_{i}\right|}{\left|y_{i}\right|}$$

MSE is the average of the squared errors between the predicted and actual values. It amplifies larger errors by squaring them, making it sensitive to outliers. A lower MSE indicates a higher accuracy, revealing that the model’s predictions are close to the actual values and vice versa. MSE is represented by

(12) $$MSE=\frac{1}{n}\sum_{i=1}^{n}{\left(y_{i}-{\hat{y}}_{i}\right)}^{2}$$

RMSE is the square root of the MSE, representing the average of the square roots of the errors between the predicted and actual values. Because RMSE involves squaring the errors before averaging, it gives more weight to larger errors, making it sensitive to outliers. The square root then brings the units of RMSE back to the same scale as the original data, making it easier to interpret in the context of the specific problem. Like MSE, its lower value indicates a higher accuracy, revealing that the model’s predictions are close to the actual values and vice versa. RMSE is calculated by

(13) $$RMSE=\sqrt{\frac{1}{n}\sum_{i=1}^{n}{\left(y_{i}-{\hat{y}}_{i}\right)}^{2}}$$

R^2^ measures the proportion of the variance in the data explained by the model. Its value represents the explanatory power of the model, where a value closer to 1 indicates a stronger explanatory power and fits the data well, and when its value is close to 0, implying a poor forecasting effect of the model. Its calculation is expressed by

(14) $$R^{2}=1-\frac{\sum_{i=1}^{n}{\left({\hat{y}}_{i}-y_{i}\right)}^{2}}{\sum_{i=1}^{n}{\left({\bar{y}}_{i}-y_{i}\right)}^{2}}$$

### 4.2. Correlation Analysis Results

**Figure 5:**
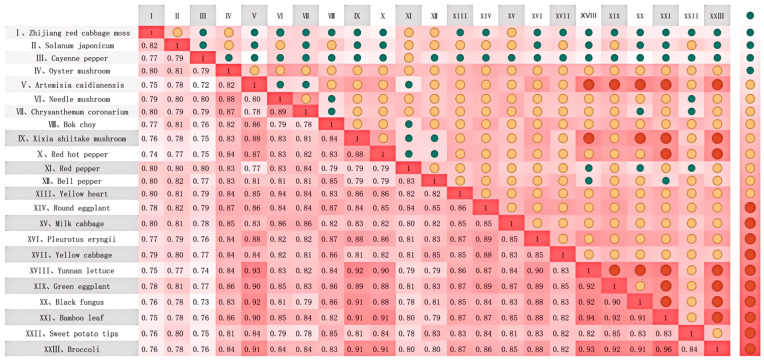
Sales correlation heatmap of short-cycle agricultural products using GRA.

The GRA is conducted to acquire the products with sales records close to the target product. This illustrates the cooperative or competitive relationships among products over time. Figure 5 describes the sales correlation heatmap of short-cycle agricultural products. It helps to extract the correlation features of the target product. For example, bamboo leaf (XXI) exhibits a strong correlation with Xixia shiitake mushroom (IX), red pepper (XI), Yunnan lettuce (XVIII), and green eggplant (XIX) in terms of sales trends. The importance of understanding such correlations to reduce food waste is widely recognized. By analyzing sales patterns and correlations between different vegetables, demand fluctuations can be better anticipated, allowing for more accurate inventory adjustments. Through a thorough understanding of the correlation coefficients between bamboo leaf sales and other products, its demand can be better predicted, and reasonable inventory adjustments can be performed. In real-world scenarios, if those correlated products experience high sales, it is advisable to stock a larger quantity of bamboo leaf. On the other hand, when the sales of these correlated products are low, reducing the inventory of bamboo leaves can prevent overstock and minimize waste. This proactive approach reduces the risks of inventory backlog and stockouts for decision-makers, optimizes supply chain management, and minimizes food waste, contributing to more sustainable and efficient practices.

Furthermore, the correlation analysis provides a basis for potential cross-product promotional activities or joint marketing efforts. By analyzing which products exhibit highly synergistic effects with bamboo leaf sales trends, decision-makers can design more precise promotion strategies. The findings of the analysis offer a deeper understanding, guiding decision-makers in selecting features to enhance model accuracy and suggesting potential correlations between bamboo leaf sales and products like green eggplant and Yunnan lettuce. Incorporating these features into models helps to explore broader food market trends and consumer preferences. Therefore, the model ensures its robustness when handling correlation features derived from potential relationships. 

**Figure 6:**
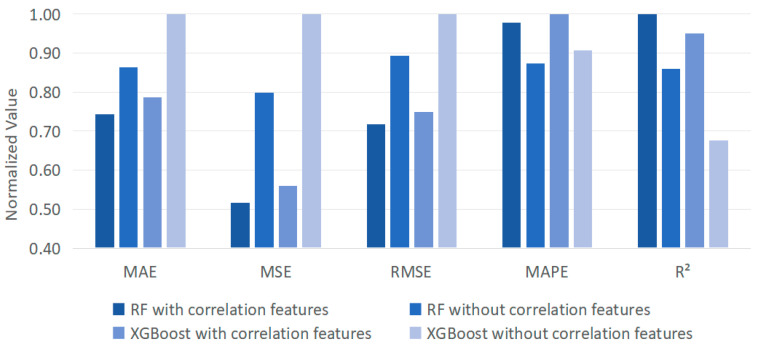
The results derived from RF and XGBoost with and without integrating the correlation features in bamboo leaf sales forecasting.

To further discuss the advance of correlation features, Figure 6 displays the results derived from RF and XGBoost with and without integrating the correction features in bamboo leaf sales forecasting. Here, data normalization shows the values of different evaluation metrics into the same range, allowing better investigation of the overall performances. The variations in Figure 6 indicate that incorporating the sales of other products correlated with bamboo leaf into the models leads to better performance in terms of MAE, MSE, RMSE, and R^2^. The reason is that relying solely on the sales data may not capture potential market-influencing factors when forecasting bamboo leaf sales. By including the sales of other highly correlated products in the model, the interplay and common trends among different products in the market can be estimated so that the prediction errors are reduced. Moreover, this enhances the understanding of the complex dynamics behind bamboo leaf sales and offers a new perspective that when predicting the sales of any product, consideration should be given to its correlation with highly correlated products. This comprehensive analytical approach enhances the forecasting capabilities of the model, which would provide appropriate guidance to achieve greater efficiency and sustainability in food distribution.

In summary, the correlation analysis shows the competition or complementarity among different short-cycle agricultural products in terms of sales, demonstrating their mutual influences and consumer preferences. It not only aids decision-makers in gaining a deeper understanding of market demand and consumption trends but also provides scientific evidence for producers to develop more accurate production plans and supply chain strategies, enhancing supply chain efficiency and optimizing market regulation methods.

### 4.3. Hierarchical Clustering Results

**Figure 7:**
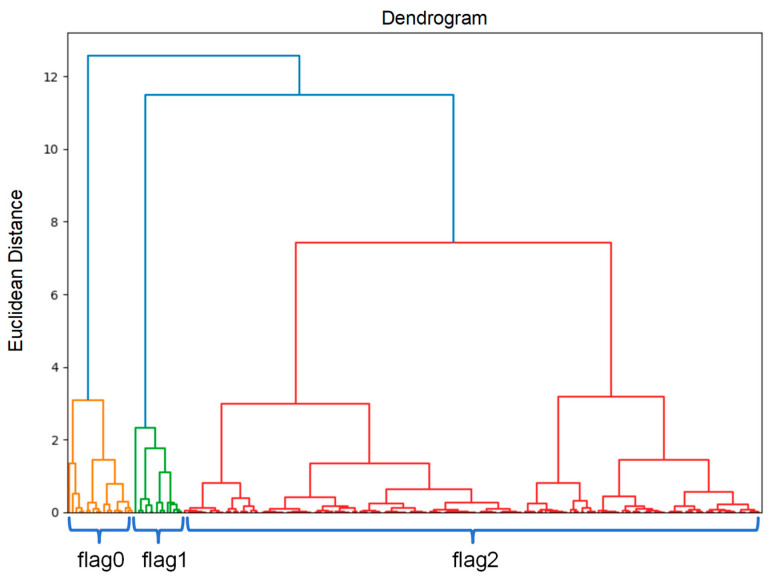
Hierarchical clustering results of Yunnan lettuce.

The hierarchical clustering produces a dendrogram as its result, and each node in the dendrogram is generated by merging a set of two member branches. The node containing all members is called the root node, while nodes representing individual original members are called leaf nodes. The positions of these leaves on the *x*-axis of the dendrogram denote their relative joining sequence, and there are various topologically equivalent ways to draw the same dendrogram, which indicates the same group relationships. On the other side, the *y*-axis represents the order of node generation with a distance. Taking Yunnan lettuce as an example, Figure 7 is the hierarchical clustering performed using the residuals from the RF-based first layer in the model.

Figure 7 displays that the residual features are relatively concentrated. Through hierarchical clustering, the residuals of daily sales are categorized into three groups: flag0, flag1, and flag2. The analysis shows that most residuals clustered around flag2, with fewer around flag0 and an even smaller portion around flag1. This categorization illustrates the correlations and differences between the various residual types, indicating distinct anomalies or error patterns in sales forecasting. Meanwhile, the hierarchical clustering results can assist in more effectively segmenting markets and allocating resources. By analyzing the characteristics of flag0, flag1, and flag2, recurring patterns in particular products, regions, or periods are identified, which can then be used to optimize supply chain management, inventory control, and sales strategies. Typically, clustering residual features allow for capturing of inherent patterns within the data. These features generated from clustering reflect underlying data structures and improve the ability to handle complex market conditions. By incorporating these clustering results as new features, the representation of sales data is enriched, providing additional context and background information. As a result, it enhances the model’s ability to integrate higher-level features and deliver more accurate forecasting results. Based on that, such representations offer more deep insights, leading to optimized management processes, and improving decision-making efficiency.

In short, the hierarchical clustering model categorizes residuals into various groups, with different labels (i.e., flag0, flag1, flag2) denoting various types of anomalies or errors in sales forecasting. This approach provides a more intuitive understanding of the distribution and changes in residuals, identifies products or market segments with potential issues, and makes targeted adjustments and optimizations. Further analysis of such labels’ characteristics also helps to identify similar patterns within specific products, regions, or periods and benefits to enhance the precision of supply chain management. 

### 4.4. Comparative Study

**Figure 8:**
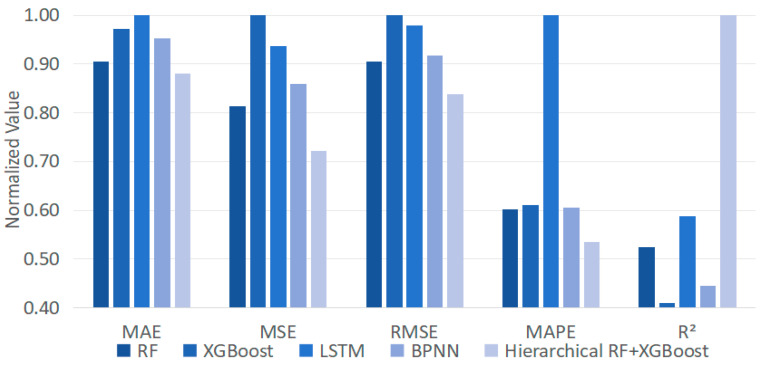
A comparison of the hierarchical RF-XGBoost model and the existing solutions.

To extensively evaluate the performance of the proposed method, a comprehensive comparative study is conducted. First, the hierarchical RF-XGBoost model is compared with the existing solutions, including the ablation experiment (i.e., standalone RF and XGBoost), LSTM, and Backpropagation Neural Network (BPNN). Based on the five evaluation metrics, the comparisons are depicted in Figure 8.

From Figure 8, the proposed method, which combines RF and XGBoost with hierarchical clustering, exhibits significant enhancement over the existing solutions across all evaluation metrics. Particularly, it demonstrates superiority over standalone RF and XGBoost, with a MAPE reduction of 10% and 12%, respectively, and an R^2^ increase of 22% and 24%, respectively. Such findings reveal that the proposed method combines the strengths of both RF and XGBoost. By sequentially linking these two models, not only can their respective advances be fully utilized, such as good at processing high-dimensional features with complex interaction relationships and effective at handling nonlinear and heterogeneous data, but their shortcomings can also be complemented, resulting in better performance. 

**Figure 9:**
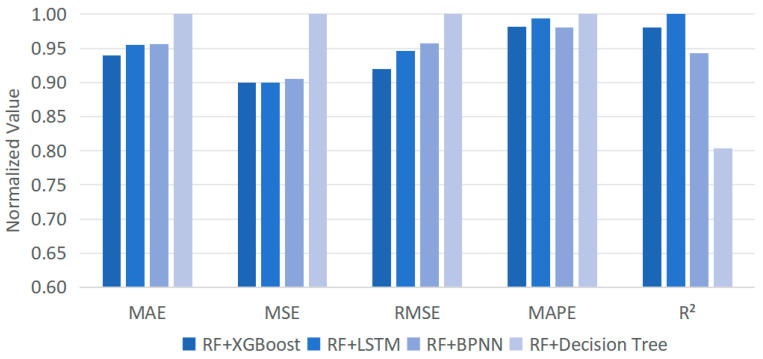
A comparison of the proposed model and the other classification methods.

Subsequently, to demonstrate the appropriate combination of RF and XGBoost, the RF is settled as the base to generate residuals, which are then employed as the inputs to other prediction models (LSTM, BPNN, and decision tree) for the same prediction task. After training and testing under the same conditions (i.e., 60% for training and 40% for testing), the results are depicted in Figure 9.

By comparing the evaluation metrics of different methods in Figure 9, the strengths and weaknesses of each model in short-cycle agricultural product sales forecasting are investigated. Overall, the proposed model yields the best performances compared with the others, except R^2^. Such results reveal that the RF is suitable to act as the base for extracting trends from the input data and passing residual information to other models for further learning and prediction. In addition, to further find the advance of RF-XGBoost, the runtime for each model is recorded, as summarized in Table 2.

The runtime results presented in Table 2 reveal insights into model complexity and computational cost. The decision tree-based method offers the shortest runtime, attributed to the simple structure and intuitive logic. It partitions data with simple conditional statements step by step, demonstrating faster speed. Then, the runtime of the proposed method is the second fastest. Although ensemble learning methods utilize multiple decision tree models, they employ different strategies in constructing each tree, where RF constructs trees by randomly selecting features, and XGBoost improves the model gradually using gradient boosting, making their computational complexity slightly higher than that of a single decision tree but still more efficient compared to LSTM and BPNN. LSTM and BPNN yield a longer running time. The reason is that both neural network models have complex structures and training processes [35]. LSTM is derived from the recurrent neural network with gate mechanisms and memory units designed for handling time-sequential data, making its training and prediction processes relatively time-consuming [36]. BPNN is a fully connected feedforward neural network that requires computation and updating all connections, resulting in longer running time, especially on large-scale datasets [37].

From this comparative study, it can be said that the proposed RF-XGBoost model shows better adaptation to the time-series complexity of data, with an impressive runtime. In real-world scenarios, there are often multiple features and relationships that conventional models may not fully capture in a time-saving manner. In this case, the proposed model combines various learners to well-suite the data variability with less time cost. Therefore, it enhances the forecasting robustness in short-cycle agricultural product sales.

### 4.5. Discussion

First, regarding the limitations of existing models, XGBoost efficiently captures nonlinear features in structured data and regression problems, but its ability to automatically detect trends and seasonality in time series data is limited, and it is sensitive to missing data. RF is robust in dealing with nonlinear data and is resistant to overfitting. However, as the number of features increases, its computational cost rises, and it struggles to process time series data with limited interpretability automatically. ARIMA model is a classic time series analysis tool that is particularly suitable for analyzing and predicting linear data exhibiting clear cycles and trends. Nonetheless, when dealing with nonlinear and highly volatile data, particularly in short-term agricultural product sales, the predictive accuracy of ARIMA may be inferior to that of advanced nonlinear models. The decision tree has a simple structure and is easy to interpret. In agricultural product sales forecasting, the decision tree can rapidly identify time-related features. Nonetheless, a single decision tree is prone to overfitting, especially when dealing with short-cycle data with high noise levels. Moreover, decision trees have limited capability in capturing long-term dependency and trend change in time series data. In this context, LSTM, a type of recurrent neural network, excels at capturing long-term dependencies in time series data. It is particularly suitable for processing agricultural product sales data characterized by seasonality and trend changes, especially in cases with short time intervals and frequent fluctuations. Although LSTM can effectively handle complex temporal dependencies, it requires a large dataset, has long training times, involves complex parameter tuning, and is prone to overfitting. Therefore, in short-term sales forecasting with smaller datasets, the advantages of LSTM may not be as significant. The BPNN updates weights through the backpropagation algorithm and is appropriate for solving nonlinear problems. However, it is not well-suited for handling time series data because it lacks a time-dependency mechanism and is prone to getting stuck in local optima. When training on large-scale data, BPNN is slower compared to more optimized models. To solve the limitations of individual models and better adapt to the dynamic and fluctuating nature of daily market demand for short-cycle agricultural products, this work proposes a hierarchical clustering model using RF-XGBoost. Such a model can be better suited to the dynamic and fluctuating nature of agricultural sales data, leading to improved forecasting accuracy. 

Second, during data preprocessing, discounts were excluded due to their typically short-term and highly volatile nature, which can cause extreme fluctuations in sales. For instance, supermarkets often discount unsold vegetables before closing or offer promotions on damaged produce during transportation and storage. Including discount factors in the forecasting model could lead to interference from short-term fluctuations, making it difficult to accurately capture the true market demand and sales trends. By excluding discounts, the model focuses more on core factors such as supply–demand relationships, seasonality, and production, which can enhance prediction stability and better reflect the essence of the market. In addition, removing discount factors reduces reliance on extra variables and simplifies the model, which helps improve model training efficiency and reduces the risk of overfitting, particularly in short-cycle forecasts. Excluding discounts allows the model to concentrate on long-term trends, which aids decision-makers in developing more strategic and sustainable production and sales plans. But, the limitation of excluding discounts is evident, as discounts usually influence consumer behavior. Ignoring them may result in the model failing to fully reflect consumer purchasing behavior in promotional situations. Hence, such key factors will be considered in future modeling.

Third, the proposed RF-XGBoost model demonstrates superior performance in prediction accuracy and stability when applied to short-term agricultural product sales data. If longer-term products with more pronounced seasonal patterns and trends are selected, it is anticipated that models like ARIMA or LSTM might become more suitable for capturing these long-term dependencies, potentially requiring adjustments to the model structure and hyperparameters to accommodate these cycles [38]. The reduced frequency of fluctuations in long-term product sales might ease the model’s burden in handling short-term noise but would likely necessitate more complex time series processing to obtain slow-moving trends, altering the balance of computational resources and training time. Besides, the impact of external factors, such as weather, market conditions, and macroeconomic trends, could be more significant over extended product cycles, making it crucial to incorporate these factors into the model. Therefore, after adjustments, the current model could also be effective for long-term forecasting, and future work will explore these adaptations for a broader range of agricultural products with various terms.

Next, the outbreak of the COVID-19 pandemic at the end of 2019 had a significant impact on industries worldwide, particularly agriculture and food market. In this work, the agricultural product sales data of a supermarket from July 1, 2020, to June 30, 2023, is used, covering the periods before and after the pandemic, which may bring unpredictable impacts on the stability and accuracy of the results. Specifically, due to labor shortages caused by pandemic restrictions, there is a reduced availability of manpower for harvesting and transporting fresh produce [39]. This disruption in the supply chain for many foods, such as fruits and vegetables, leads to unavoidable price volatility. Price fluctuations often influence consumer purchasing decisions. However, short-cycle agricultural products, as essential daily food items, have relatively inelastic demand. When the price of a particular vegetable rises sharply, consumers typically opt for substitutes or reduce their purchase volume but rarely stop consumption entirely [40]. This substitution effect reflects consumers’ flexible choices in response to price increases. To analyze the specific impact of this phenomenon on sales, the correlation analysis is adopted to study the competitive and complementary relationships among different short-cycle products sold on the same day, and such factors are incorporated into the model variables. In addition, through multi-layered processing, it conducts multiple forecasts on sales, allowing for a more exhaustive decomposition and analysis of the complex structures in the data. In this regard, the proposed model aims to minimize the interference of the pandemic on forecasting results, but unfortunately, the market uncertainties brought by the pandemic may still retain an unavoidable influence.

Finally, by analyzing the competitive and synergistic effects of sales between various short-cycle agricultural products, a foundation for optimizing supply chain management and sales strategies can be established. The validation adopts five key metrics to quantitatively evaluate the forecasting model, demonstrating its ability to accurately capture sales fluctuations with high prediction. Lower MAE and RMSE values indicate that the predicted sales values closely align with actual sales, assisting decision-makers in better understanding market demand, optimizing resource allocation, and formulating effective policies. MAPE provides relative error insights, making it easier to evaluate the market performance of various agricultural products. A high R^2^ value reflects the model’s strong explanatory power regarding market fluctuations, aiding in addressing potential market risks. Thus, accurate forecasts enable producers to adjust their production plans, which can reduce the risk of overstocking and enhance supply chain management efficiency for minimizing waste. Consumers benefit from a more stable supply of agricultural products and consistent pricing, as precise sales forecasts help prevent shortages and price volatility, contributing to a more stable market environment. Such findings offer valuable insights for promoting managerial and sustainable food development.

## 5. Conclusions

To enhance the robustness of short-term agricultural product sales forecasting, a hierarchical RF-XGBoost model has been proposed. This combination is designed to mitigate the inherent randomness of a single model and apply the complementary advantages of both approaches. Regarding the performance evaluation, the proposed model has been assessed against various conditions and existing solutions. The results demonstrate that the combination of RF and XGBoost outperforms the others across the evaluation metrics, indicating superior forecasting capability in this field. Furthermore, the model costs less computational time, an additional advantage in short-term forecasting. Such performances reveal that AI addresses a research gap by effectively utilizing appropriate machine learning algorithms for sales forecasting, as it captures the fluctuation characteristics of sales more precisely. 

Although the proposed model performs well, the design of the layered structure increases the consumption of computational resources and time, and different models vary in their actual effectiveness and resource requirements. Therefore, selecting the appropriate model settings is vital for practical applications in real-world scenarios. Due to the challenges in quantifying and obtaining data for external factors, these factors are excluded in this work. Usually, external factors such as weather, policy adjustments, pandemics, market competition, and macroeconomic conditions significantly affect agricultural product sales. Consequently, one of the future investigations will focus on exploring the effects of these external factors on the hierarchical model. Besides, further exploration of model combinations will be conducted to optimize the runtime without compromising accuracy, possibly through parallel processing.

In conclusion, short-cycle agricultural product sales forecasting provides the advantages of optimizing the supply chain from producers to consumers and minimizing food waste. Thus, the proposed model aims to achieve more scientific and reliable sales predictions. Such forecasts assist in informed decision-making and strategic planning, benefiting decision-makers, producers, and consumers. Decision-makers can craft effective policies and strategies to promote sustainable development and the environment. Producers can utilize these forecasts to optimize production plans and supply chain management, satisfying market demands and reducing food waste. Consumers can plan their purchases better, avoiding future price hikes. This systematic approach enables all parties to adapt to food market changes more efficiently, reducing waste and promoting sustainable growth in agriculture. In the future, several advanced approaches [41,42,43,44] in the related fields will be investigated to enhance the generalization of the model.

## Figures and Tables

**Table 1:** The samples of short-cycle agricultural product sales data.

Category	Leafy Vegetables	Peppers	Solanacea	Edible Fungi	Aquatic Root Vegetables	Cauliflower
Agricultural product	Yellow cabbage	Red pepper	Green eggplant	Xixia shiitake mushroom	Takana vegetable	Broccoli
Sales day	904	755	845	821	159	1076
Maximum selling price (CNY/kg)	14.0	35.6	16.0	27.6	29.6	19.8
Minimum selling price (CNY/kg)	3.9	6.0	1.9	12.0	7.5	3.8
Maximum daily sales volume (kg)	187.729	113.844	34.252	79.166	11.046	152.132
Minimum daily sales volume (kg)	0.161	0.158	0.229	0.05	0.176	0.632

**Table 2:** Runtime of various models for the prediction tasks.

Model	Time (s)
RF + XGBoost	12.4
RF + LSTM	229.1
RF + BPNN	90.6
RF + Decision Tree	6.3

## Data Availability

The datasets generated and/or analyzed during the current study are available at https://github.com/fire-xian/Hierarchical-prediction (accessed on 15 July 2024).
